# Parental involvement and school engagement reduce adolescent aggressive behaviors: a three wave cross-lagged model

**DOI:** 10.3389/fpubh.2025.1455554

**Published:** 2025-05-30

**Authors:** Zhen Wang, Yuanyuan Chen, Yunsheng Su, Yan Song, Zhiyuan Tao

**Affiliations:** ^1^Guangzhou Xinhua University, Dongguang, Guangdong, China; ^2^School of Education, Guangzhou University, Guangzhou, Guangdong, China; ^3^School of Journalism and Communication, Jinan University, Guangzhou, Guangdong, China; ^4^Guangzhou Technician College, Guangzhou, Guangdong, China; ^5^School of Health Management, Guangzhou Medical University, Guangzhou, Guangdong, China

**Keywords:** parental involvement, school engagement, aggressive behaviors, cross-lagged model, adolescents

## Abstract

**Objective:**

Aggressive behaviors can have severe negative consequences on the psychological and behavioral development of adolescents. This study incorporated parental involvement, school engagement, and aggressive behaviors to construct a cross-lagged model to explore the mediating mechanism of school engagement between parental involvement and aggressive behaviors.

**Methods:**

A total of 1,835 adolescents (55.9% boys; *Mean*_age_ = 12.34) completed three rounds of offline survey questionnaires (T1–T3). Descriptive statistics were performed using SPSS 26, followed by the construction of a cross-lagged model using Mplus 8.4.

**Results:**

The structural equation model showed: (1) T2 school engagement positively mediated the relationship between T1 parental involvement and T3 aggressive behaviors; (2) A bidirectional relationship was established, where T2 school engagement also mediated the relationship between T1 aggressive behaviors and T3 parental involvement.

**Conclusion:**

This study reveals the detailed mechanisms of how parental and school engagement influence adolescent aggressive behaviors, emphasizing the importance of the interaction between individuals and their environment in the mechanisms of aggressive behaviors.

## 1 Introduction

Aggressive behaviors refers to intentional harm directed towards the physical and mental health of others, with 0.6–29.5% of global adolescents exhibiting aggressive behaviors (1). Aggressive actions not only cause physical and psychological damage to others (2, 3) but also threaten the development of the aggressor (4, 5). Engaging in aggression may relate to worse peer relationships and increases the likelihood of being targeted by aggression (6). High frequency of aggressive behaviors in adolescents is associated with poor future social adaptation (7) and academic performance (8). It also increases the risk of anxiety, depression, self-harm, and the likelihood of engaging in violent criminal activities (9–12).

Therefore, how to reduce aggressive behaviors in adolescents has become a topic of interest for psychological researchers. From the perspective of ecological systems theory (13), the family is a proximal microsystem that significantly influences adolescent behavior development (14, 15). Positive family upbringing can reduce aggressive behaviors in adolescents (15). Parental involvement is a crucial component of this, playing a vital role during adolescence (16).

Currently, there is no widely accepted definition of parental involvement (17). Broadly speaking, it refers to all behaviors by parents at home and in school that benefit their children’s education and psychological health development, including emotional, social, domestic, and educational involvement. Some researchers have categorized it into subtypes such as school-based, home-based, and academic socialization (18). Active parental involvement in a child’s education, understanding their school experiences and interpersonal relationships, can help reduce negative emotions (19) and build positive academic motivation (20). Conversely, low parental involvement in the home environment is indicative of higher internalizing and externalization problems in children (21). Generally, the consensus among researchers is that parental involvement has a positive impact (15, 21). However, whether parental involvement can continue to play a protective role in reducing aggressive behaviors in Chinese adolescents remains unanswered by empirical research.

Hendriks et al. (22) summarized 72 meta-analyses and systematic reviews, finding that parental involvement in the treatment process is one of the few factors that can suppress child aggression. A study on children in Luxembourg found that low parental involvement could only predict aggressive behaviors in girls (23). Meanwhile, a cross-sectional study on rural Chinese children examined parental involvement as a moderating variable that could mitigate the relationship between negative peer influence and aggressive behaviors and the regression analysis further revealed a significant positive association between parental involvement and aggressive behaviors (24). Another study on adolescents from diverse ethnic backgrounds found that parental involvement even intensified the relationship between exposure to violence and aggression (25). These findings are concerning. Some researchers have tried to demonstrate that parental involvement in children’s lives and education could reduce their aggressive behaviors (24). However, some results have also found negative effects of parental involvement (25). Moreover, most current studies are cross-sectional and do not adequately focus on Chinese adolescents. This study aims to fill this gap by focusing on adolescents, a group prone to aggressive behaviors, and further verifies the relationship between parental involvement and adolescent aggressive behaviors using a three-wave longitudinal sample.

### 1.1 The mediating role of school engagement

Overall, parental involvement might act as a protective social resource to reduce aggressive behaviors in adolescents, but the underlying mechanisms require further consideration. From the perspective of ecological systems theory, the family and school systems are two key microsystems during adolescence, jointly influencing the individual development of adolescents. Thus, considering school-related factors is crucial. Parental involvement significantly affects the level of school engagement among students (26, 27), and school engagement has also been found to act as an indirect protective factor against aggressive behaviors in adolescents (28). Adolescents who actively participate in school activities are more likely to adhere to school norms, values, expectations, and regulations, which reduces their risks of behavior problems (29). This study includes school engagement to verify its mediating mechanism between parental involvement and adolescent aggressive behaviors.

School engagement encompasses three aspects: cognitive, emotional, and behavioral. Cognitive involvement refers to an individual’s engagement with academic tasks, the learning process, and motivation; emotional involvement relates to students’ emotional responses and their sense of belonging to the school; behavioral involvement refers to the extent to which students actively participate in academic activities, on-campus social interactions, and extracurricular activities (30, 31). Adolescents with high school engagement are more motivated to engage in academic tasks, participate in school environment activities, and feel a greater sense of belonging and connection to the school (31). School engagement is unique as it is influenced by the student’s own cognition, motivation, emotional state, as well as by teachers, peers, and the learning environment. Constructing the “parental involvement - school engagement - aggressive behaviors” model clarifies how adolescents’ proximal system (family) affects their future interactions with other systems (school) and whether it ultimately impacts their aggressive behaviors.

It is not a new topic to think about how to reduce the aggressive behavior of teenagers from the perspective of family and school. However, with the rapid development and change of the current environment in China (e.g., the parenting perspectives still impacted by Western educational concepts, the high stressed school context), it is very necessary to pay attention to this theme continuously. Present study will use three waves of longitudinal data, a cross-lagged model is constructed to prove two main hypotheses:

*H1:* Parental involvement at T1 and T2 can significantly predict the level of adolescent aggressive behaviors at T2 and T3, respectively.

*H2:* School engagement at T2 will significantly mediate the relationship between parental involvement at T1 and adolescent aggressive behaviors at T3.

**Figure 1:**
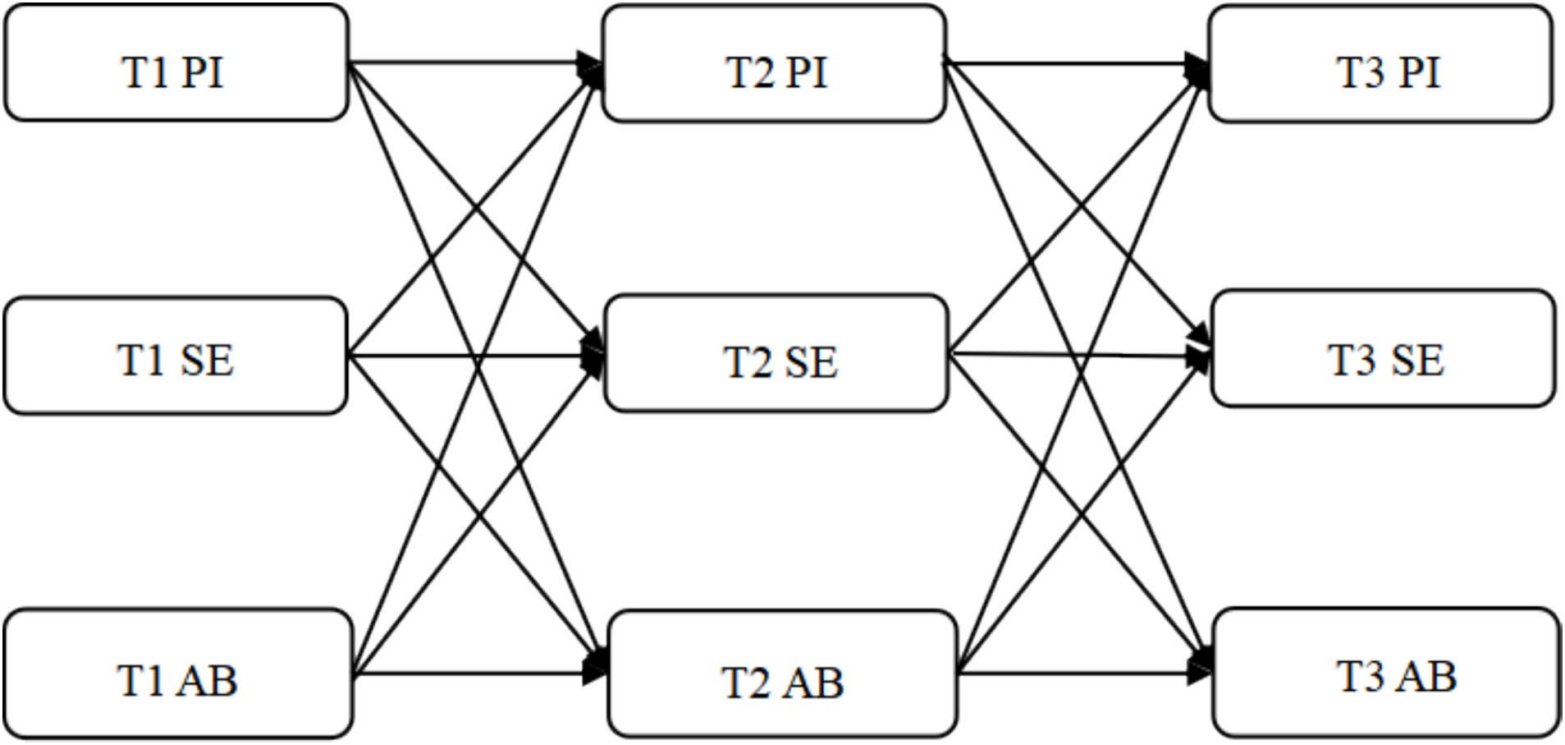
Theoratical model. PI, parental involvement; SE, school engagement; AB, aggressive behaviors.

Lastly, we want to add that we do not deny that adolescent aggressive behaviors might have a reverse impact on parental involvement. In fact, there could be a bidirectional dynamic process where aggressive behaviors not only are influenced by family and school factors but may also, in turn, affect the structure and function of these microsystems (The theoretical model is detailed in Figure 1).

## 2 Methods

### 2.1 Sample

This study recruited middle school students from three middle schools in Guangdong and Shandong provinces in China. There were no significant differences in research variables and demographic variables between students from the two provinces. Data from all participants were collected every 6 months during their transition from seventh to eighth grade. The sample include 1,835 adolescents (55.9% boys; T1 *Mean*_age_ = 12.34, *SD* = 0.52), reflecting the demographic structure of adolescents in that region, with 71.7% of families having a monthly income over 3,000 yuan. Data were collected in four waves (Time 1–Time 3), each 6 months apart: fall of 7th grade, spring of 7th grade and fall of 8th grade. At T1 (Time 1), a total of 1,835 adolescents participated in the baseline assessment. T2 (Time 2), 1,673 adolescents (91.17% of the original sample; 55.8% male) participated in the assessment. T3 (Time 3), 1,681 adolescents (91.61% of the original sample; 55.5% male) participated in the assessment. There were no significant differences in the used variables between demographic variables between the final retained sample and dropped out sample. The study was approved by the Research Ethics Committee of the corresponding author’s institution.

### 2.2 Measurements

#### 2.2.1 Parental involvement

We measured parental involvement using a perceptions of parents scale (32), which shown good reliability and validity in past research of Chinese adolescents (33). This scale is divided into two subscales (father and mother components), measuring parents’ multifaceted attention towards their children’s academic performance, behavioral patterns, thought processes, and developmental issues, including whether they pay attention to their children’s school life, frequently communicate with them, and are aware of their daily activities. Adolescents reported the frequency of parental involvement in their learning and life over the past 6 months (e.g., “Father wants to know what I am doing,” “Mother likes to talk with my teacher about my school life”). The scale was rated using a 4-point Likert scale, and the final score was the sum of three item scores, with higher scores indicating higher levels of parental involvement. In this study, the Cronbach’s alpha values at T1, T2, and T3 were 0.865, 0.879, and 0.892, respectively.

#### 2.2.2 School engagement

School engagement was measured using a 23-item school engagement scale initially developed by Wang et al. (34), which shown good reliability and validity in past research of Chinese adolescents (35). Respondents were asked to describe their behavior (e.g., “How often do you skip classes?”), emotional (e.g., “I feel happy and safe while in school”), and cognitive engagement (e.g., “Receiving a good education is the best way to succeed in life”) with school. The items were rated using a 5-point Likert scale. Scores were averaged, with higher scores indicating higher levels of school engagement. In the current study, the Cronbach’s alpha values at T1, T2, and T3 were 0.797, 0.809, and 0.833, respectively.

#### 2.2.3 Aggressive behaviors

Aggressive behaviors was measured using 19-item Chinese version of the Buss-Warren aggression questionnaire (BWAQ) (36), which shown good reliability and validity in past research of Chinese adolescents (37). The scale was scored on a 5-point scale, from 1 (never) to 5 (always). The children’s aggressive behaviors toward others were assessed, including verbal aggression (e.g., speaking ill of others behind their backs) and physical aggression (e.g., hitting others). Scores for aggressive behaviors were averaged, with higher scores indicating a higher frequency of initiating aggressive behaviors. In this study, the Cronbach’s alpha values at T1, T2, and T3 were 0.862, 0.874, and 0.885, respectively.

### 2.3 Statistical analysis

Descriptive statistics were performed using SPSS 23.0. A cross-lagged structural equation model was constructed using Mplus 8.4. During conducting formal statistical analysis, missing data were handled using full information maximum likelihood estimation, and all results were standardized. Bootstrap was used to test and validate the statistical significance of pathways and indirect effects in each model. Multiple fit indices were used to assess model fit, including the chi-square to degrees of freedom ratio (*χ*^2^/*df*), Comparative Fit Index (CFI), and Root Mean Square Error of Approximation (RMSEA).

## 3 Results

### 3.1 Descriptive statistics

The results showed that parental involvement at T1 was negatively correlated with school engagement at later time points and positively correlated with aggressive behaviors. School engagement at T1 time point was negatively correlated with aggressive behaviors at later time points (The correlative results are detailed in Table 1). The correlations between variables match the present hypothesis. And the regression model showed that parental involvement and school engagement could predict the next wave aggressive behaviors (The regression models are detailed in Table 2). Regression results showed that parental involvement could predict the next wave school engagement and school engagement could predict the next wave aggressive behaviors.

**Table 1:** Means, standard deviations, and Pearson correlation coefficients of all variables.

Variables	1	2	4	5	6	7	8	9	10	11
1. Gender	–									
2. T1 Age	−0.03	–								
3. T1 PI	−0.09***	−0.08***	**–**							
4. T2 PI	−0.13***	−0.06*	0.64***	**–**						
5. T3 PI	−0.11***	−0.08***	0.55***	0.66***	–					
6. T1 SE	0.04	−0.03	0.38***	0.33***	0.26***	–				
7. T2 SE	0.04	−0.02	0.31***	0.40***	0.36***	0.61***	–			
8. T3 SE	0.06*	−0.04	0.27***	0.34***	0.39***	0.54***	0.67***	–		
9. T1 AB	−0.04	−0.01	−0.16***	−0.11***	−0.09***	−0.34***	−0.28***	−0.24***	–	
10. T2 AB	−0.04	0.01	−0.08***	−0.09***	−0.07***	−0.25***	−0.31***	−0.28***	0.43***	–
11. T3 AB	−0.05*	−0.01	−0.06*	−0.09***	−0.10***	−0.22***	−0.25***	−0.30***	0.42***	0.49***
Mean	1.44	12.34	2.88	2.78	2.85	4.00	3.84	3.79	1.20	1.21
SD	0.50	0.52	0.65	0.67	0.68	0.57	0.60	0.62	0.41	0.41

Bold values: significant correlations. Gender is a dummy variable, *n* = 1,007, 1 = boy, 0 = girl; PI, parental involvement; SE, school engagement; AB, aggressive behaviors. *p < 0.05, ***p < 0.001.

**Table 2:** The regression models results.

Predict variables	*β*	*SE*	*t*	*β*	*SE*	*t*
	Equation 1—T2 School engagement			Equation 2—T2 Aggressive behaviors		
Gender	0.01	0.03	0.06	−0.01	0.02	−0.21
Age	0.01	0.03	0.19	0.01	0.02	0.38
T1 Parental involvement	0.09***	**0.03**	**4.01**	0.02	0.02	0.84
T1 School engagement	0.57***	**0.02**	**24.66**	−0.13***	**0.02**	**−4.81**
T1 Aggressive behaviors				0.38***	**0.03**	**15.33**
	Equation 3—T3 School engagement			Equation 4—T3 Aggressive behaviors		
Gender	0.05*	**0.03**	**2.26**	−0.05*	**0.02**	**−2.23**
Age	−0.03	0.03	−1.36	−0.01	0.02	−0.14
T2 Parental involvement	0.08***	**0.02**	**3.69**	−0.02	0.02	−0.70
T2 School engagement	0.64***	**0.02**	**28.23**	−0.11***	**0.02**	**−4.12**
T2 Aggressive behaviors				0.47***	**0.02**	**18.28**

Bold values: significant correlations. *p < 0.05, ***p < 0.001.

A further construction of cross-lagged models to verify relationships. The structural equation model analysis of parental involvement, school engagement, and aggressive behaviors using three waves of data showed, *χ*^2^ = 4.88, *df* = 6, *χ*^2^/*df* = 0.81, CFI = 1.00, TLI = 1.00, RMSEA = 0.001, SRMR = 0.004, indicating an excellent model fit.

**Figure 2:**
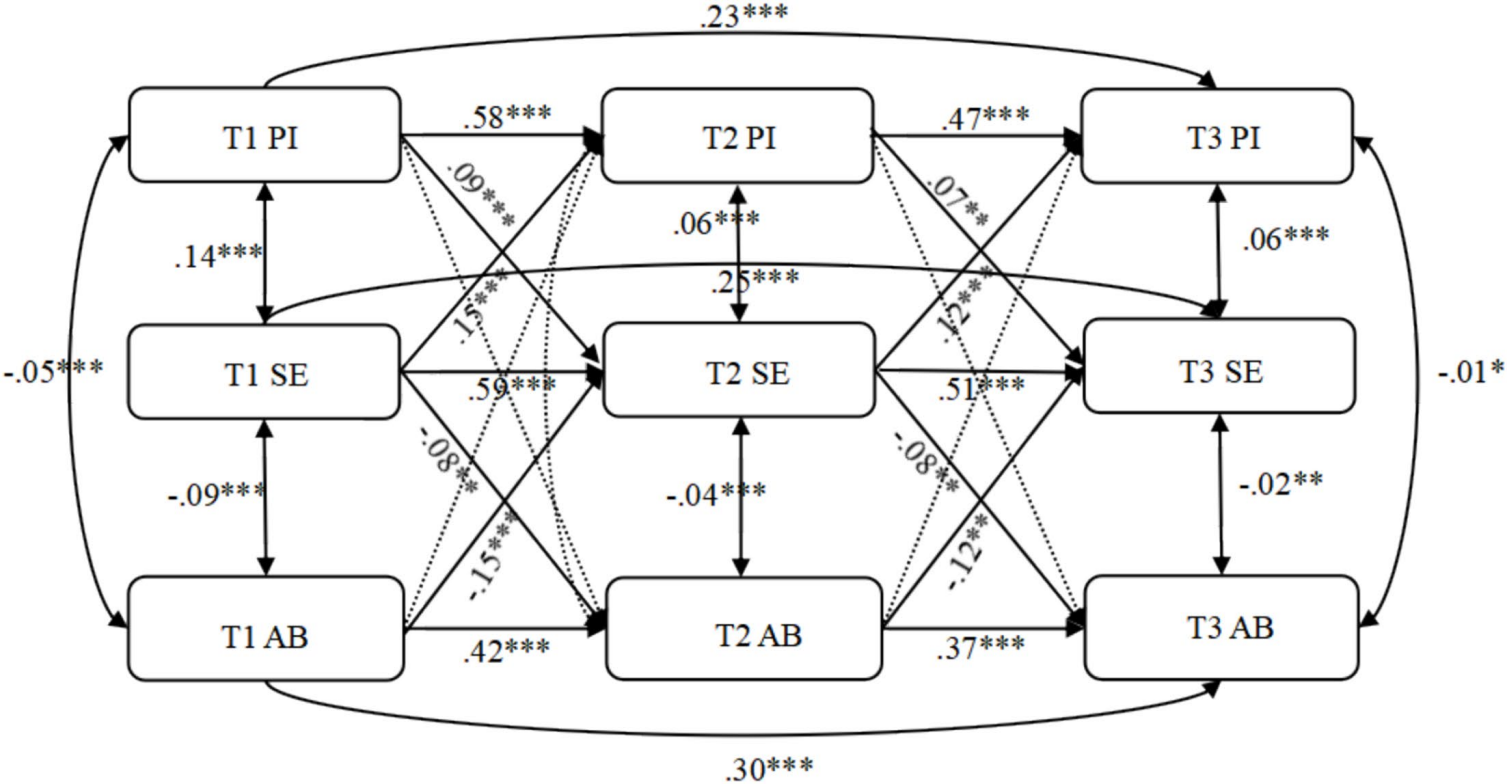
Cross-lagged model results. *p < 0.05, **p < 0.01, ***p < 0.001; PI, parental involvement; SE, school engagement; AB, aggressive behaviors. Dashed lines represent insignificant paths.

All auto regressive paths in the cross-lagged models were significant, as were the bidirectional correlation paths within the same time points (The correlative results is detailed in Figure 2). Figure 2 in the cross-lagged path results showed that T1 parental involvement positively predicted T2 school engagement (*β* = 0.09, *p* < 0.001), and T2 school engagement significantly negatively predicted T3 aggressive behaviors (*β* = −0.08, *p* < 0.01). The mediating path “T1 parental Involvement → T2 school engagement → T3 aggressive behaviors” was established (*β*_indirect_ = −0.01, *p* < 0.05). Parental involvement cannot directly predict next time point aggressive behaviors.

Additionally, it should be noted that the reverse indirect pathway in the cross-lagged model was also significant. T1 aggressive could negatively predict T2 school engagement (*β* = −0.15, *p* < 0.001) and T2 school engagement could positively predict T3 parental involvement (*β* = 0.12, *p* < 0.001). The mediating path “T1 parental Involvement → T2 school engagement → T3 aggressive behaviors” was established (*β*_indirect_ = −0.01, *p* < 0.01). Aggressive behaviors also cannot directly predict next time point parental involvement. In all the time pointed, parental involvement show positive relationship with school engagement and school engagement show negative relationship with aggressive behaviors.

## 4 Discussion

This study, utilizing a longitudinal latent cross-lagged structural equation model, meticulously validated the relationships among parental involvement, school engagement, and aggressive behaviors within a Chinese adolescent population. Both hypotheses proposed by the study were confirmed, addressing gaps in existing research. The results highlighted the bidirectional relationship between parental involvement and aggressive behaviors, revealing complex mechanisms between the variables.

Firstly, parental involvement was found to negatively correlated to adolescent aggressive behaviors. This could be attributed to the emotional support included in parental involvement, providing adolescents with a secure environment where they can freely express emotions and concerns (38), thereby reducing the likelihood of expressing discontent through aggressive behaviors. Parents, by communicating with schools and understanding their children’s academic and behavioral performance, can accurately gauge the adolescents’ status at school. Based on this, parents can communicate with their children, set common expectations and boundaries, and appropriately monitor and guide adolescent behavior (39, 40), which helps reduce occurrences of aggression. When adolescents are aware that their actions are closely monitored by their parents and that aggressive behaviors will have negative consequences, they are more likely to adhere to rules. However, in the cross-wave regression analysis, parental involvement failed to predict adolescent aggressive behaviors at later time points. This may suggest that parental influence on adolescents during puberty is weaker compared to school environmental factors. Alternatively, it could be interpreted that the triggers for adolescent aggression are more likely to originate in school settings, such as the school engagement in our research. The other factors, for example, observational learning of aggressive behaviors from deviant peers, impulsive acts of aggression, or verbal/relational bullying to maintain social status within peer groups (41). Furthermore, the significant effects observed in the regression model indicate that parental involvement may indirectly shape adolescent aggression by influencing other microsystems (e.g., family dynamics or community interactions). Thus, these findings do not diminish the role of parental involvement in promoting positive adolescent development.

Additionally, school engagement was found to negatively mediate the relationship between parental involvement and adolescent aggressive behaviors. The positive relationship between parental and school engagement underscores the positive impact of proactive family upbringing on children’s participation in school activities, building of learning motivation, and establishing interpersonal relationships with classmates and teachers (42). Parents actively understanding adolescents’ school life can cultivate a positive learning attitude, enhancing children’s engagement in school activities. Parents’ expectations, supervision, and academic support (such as helping with homework and discussing academic content) directly increase students’ focus and participation in the classroom. Active parental interest and support in adolescents’ academics increase their interest and commitment to school. Adolescents will perceive school as an important and worthwhile place to invest in. This emotional investment establishes positive connections between children and school, enhancing their sense of belonging and satisfaction (43). When parents communicate with schools and align on educational goals and values, children receive consistent messages from both critical environments, aiding in their understanding of the importance of education and performing better at school. These consistent messages can reduce cognitive conflicts felt by children, enabling them to focus more on their studies (44). Moreover, effective communication between parents and teachers can help teachers better understand and support students’ individual needs. Parental involvement also helps cultivate adolescents’ self-control, sense of responsibility, and social skills (45–47). These skills are crucial components of school success and can foster positive school engagement among students.

High school engagement can significantly predict lower aggressive behaviors. Schools provide a social environment where adolescents with high school engagement actively invest emotionally and learn how to interact with peers. This helps them use adaptive methods rather than aggression to handle interpersonal conflicts (31). A strong sense of belonging and positive relationships with classmates and teachers also provide social support to adolescents, reducing feelings of isolation and thereby decreasing aggressive behaviors (43). Students with high levels of school engagement are also more likely to focus on their studies. This focus can divert their attention away from boredom or frustration (48, 49), which are common triggers for aggressive behaviors. If adolescents actively participate in sports, music, or other team activities, they can also develop teamwork and social skills. These skills enable students to handle conflicts more effectively and reduce the likelihood of using aggressive behaviors to solve problems.

Furthermore, our results revealed a reverse pathway from aggressive behaviors to both school and parental involvement. Adolescents who exhibit aggressive behaviors may face peer rejection, which can reduce their emotional involvement and social interactions at school (50). A lack of peer support and social networks can lower their satisfaction with school and sense of belonging, further affecting emotional involvement. Aggressive behaviors might lead to negative perceptions and expectations from teachers and school administrators, thereby affecting the academic support and opportunities available to students. Moreover, due to possible school disciplinary measures (such as suspension or other punishments) related to behavioral issues, students may miss important learning opportunities, affecting their cognitive involvement. Schools might limit adolescents with a history of aggressive behaviors from participating in certain activities like sports or clubs to avoid potential conflicts. This restriction reduces their opportunities for behavioral involvement, affecting their holistic development in school.

Children’s aggressive behaviors may increase parental stress and anxiety. Dealing with children’s behavioral issues, parents may feel powerless or frustrated, which can affect how they interact with and participate in school activities (51). To address their children’s behavioral issues, parents may need to spend more time and resources at home, potentially reducing their involvement in school activities. Furthermore, if parents feel that their educational methods have failed to improve their children’s behavior, they might become discouraged, thus reducing cooperation with the school (52). Aggressive behaviors can lead to increased tension between the family and school, especially when dealing with disciplinary issues. If parents are dissatisfied with how the school handles these issues, it could affect their communication and cooperation with the school, reducing positive parental involvement (53).

In conclusion, our results clearly reveal the complex relationships between parental involvement, school engagement, and adolescent aggressive behaviors. These results align well with the assumptions of ecological systems theory. Parental and school engagement, as two crucial microsystems, interact to form a mesosystem that influences adolescent behavioral development. The individual in turn impacts the surrounding environment, creating a feedback loop. In this cycle, the level of parental involvement is likely the initial influencing factor. Therefore, we emphasize the importance of parental involvement. From the time children first enter the school environment, parents should actively participate in their lives. Increasing communication with educational staff and understanding children’s behavior at school ensures that aggressive behaviors can be monitored promptly (53). If parental involvement was lacking during kindergarten and elementary stages, our findings suggest that enhancing involvement from the middle school stage can also curb the development of aggressive behaviors. As researchers, we hope parents recognize the necessity of being involved in their children’s school life, reducing the risk of initiating aggressive behaviors from the earliest stages.

This study contributes both theoretically and practically by employing three-wave longitudinal data to rigorously explore the temporal relationships between parental involvement, school engagement, and aggressive behaviors (54). The findings validate the theoretical framework of ecological systems, demonstrating that family systems influence school systems, which in turn shape the developmental trajectory of aggressive behaviors. Our results suggest that intervention researchers should prioritize school-system factors when addressing adolescent aggression. Specifically, school engagement emerged as a stronger direct predictor of aggressive behaviors compared to parental involvement, highlighting the critical role of educational environments in behavioral interventions.

## 5 Limitations

While this study has made theoretical contributions based on longitudinal data, there remain limitations that warrant further refinement. Peer influence is one of the strongest predictors of adolescent behavior, underscoring the importance of including peer-related variables as covariates. Additionally, incorporating other relevant covariates could further enhance the robustness of the findings. Furthermore, existing research on adolescent aggressive behavior predominantly focuses on aggression directed at peers, as does this study. This emphasis aligns with the fact that peer-directed aggression is the most prevalent form among adolescents, and addressing this issue could broadly reduce its incidence. However, cutting-edge research has begun exploring adolescent aggression toward parents, particularly behaviors driven by oppositional defiance (55, 56). Although such behaviors occur less frequently, integrating observational measures of these phenomena in future studies would provide a more comprehensive understanding of the development of adolescent aggression.

## 6 Conclusion

This study found that parental involvement can significantly positively predict future levels of adolescent aggressive behaviors. School engagement can negatively mediate the path from parental involvement to adolescent aggressive behaviors, as well as the path from adolescent aggressive behaviors to parental involvement. Such findings emphasize the interaction among adolescents, families, and schools, collectively influencing the development of aggressive behaviors in adolescents.

## Data Availability

The raw data supporting the conclusions of this article will be made available by the authors without undue reservation.
